# *KIR*, *HLA*, and *IL28B* Variant Predict Response to Antiviral Therapy in Genotype 1 Chronic Hepatitis C Patients in Japan

**DOI:** 10.1371/journal.pone.0083381

**Published:** 2013-12-12

**Authors:** Yuichi Nozawa, Takeji Umemura, Satoru Joshita, Yoshihiko Katsuyama, Soichiro Shibata, Takefumi Kimura, Susumu Morita, Michiharu Komatsu, Akihiro Matsumoto, Eiji Tanaka, Masao Ota

**Affiliations:** 1 Division of Hepatology and Gastroenterology, Department of Medicine, Shinshu University School of Medicine, Matsumoto, Japan; 2 Department of Pharmacy, Shinshu University Hospital, Matsumoto, Japan; 3 Department of Legal Medicine, Shinshu University School of Medicine, Matsumoto, Japan; University of Sydney, Australia

## Abstract

Natural killer cell responses play a crucial role in virus clearance by the innate immune system. Although the killer immunoglobulin-like receptor (KIR) in combination with its cognate human leukocyte antigen (HLA) ligand, especially *KIR2DL3-HLA-C1*, is associated with both treatment-induced and spontaneous clearance of hepatitis C virus (HCV) infection in Caucasians, these innate immunity genes have not been fully clarified in Japanese patients. We therefore investigated 16 KIR genotypes along with *HLA-B* and *-C* ligands and a genetic variant of interleukin (IL) 28B (rs8099917) in 115 chronic hepatitis C genotype 1 patients who underwent pegylated-interferon-α2b (PEG-IFN) and ribavirin therapy. *HLA-Bw4* was significantly associated with a sustained virological response (SVR) to treatment (*P* = 0.017; odds ratio [OR] = 2.50, ), as was the centromeric A/A haplotype of *KIR* (*P* = 0.015; OR 3.37). In contrast, SVR rates were significantly decreased in patients with *KIR2DL2* or *KIR2DS2* (*P* = 0.015; OR = 0.30, and *P* = 0.025; OR = 0.32, respectively). Multivariate logistic regression analysis subsequently identified the *IL28B* TT genotype (*P* = 0.00009; OR = 6.87, 95% confidence interval [CI] = 2.62 - 18.01), *KIR2DL2/HLA-C1* (*P* = 0.014; OR = 0.24, 95% CI = 0.08 - 0.75), *KIR3DL1/HLA-Bw4* (*P* = 0.008, OR = 3.32, 95% CI = 1.37 - 8.05), and white blood cell count at baseline (*P* = 0.009; OR = 3.32, 95% CI = 1.35 - 8.16) as independent predictive factors of an SVR. We observed a significant association between the combination of *IL28B* TT genotype and *KIR3DL1*-*HLA-Bw4* in responders (*P* = 0.0019), whereas *IL28B* TT along with *KIR2DL2-HLA-C1* was related to a non-response (*P* = 0.0067). In conclusion, combinations of *KIR3DL1/HLA-Bw4*, *KIR2DL2/HLA-C1*, and a genetic variant of the *IL28B* gene are predictive of the response to PEG-IFN and ribavirin therapy in Japanese patients infected with genotype 1b HCV.

##  Introduction

 Hepatitis C virus (HCV) infection is a major cause of chronic liver disease worldwide. Chronic HCV infection often develops into chronic hepatitis, which may progress to liver cirrhosis and/or hepatocellular carcinoma (HCC)[1]. HCC is a leading cause of death from malignant neoplasms in Japan[2]. Since approximately 70% of Japanese HCC patients are infected with HCV, the successful eradication of this virus, defined as a sustained virological response (SVR), is considered important to decrease the incidence of HCC. 

Natural killer (NK) cells are key components of the innate antiviral immune response that are controlled by a balance of activation and inhibitory receptors. NK cell activation receptors include C-type lectin-like receptors (NKG2C, NKG2D, and NKG2E), natural cytotoxicity receptors (NKp30, NKp44, and NKp46), and CD16, while known inhibitory receptors include killer cell immunoglobulin-like receptors (KIRs) and the CD94/NKG2 family, which also contains a C-type lectin-like receptor (NKG2A) [3,4]. Sixteen *KIR* genes and pseudogenes have been identified that are encoded by a family of genes located on human chromosome 19q13.4. One particular feature of *KIRs* is their substantial genetic diversity. Some inhibitory *KIR*s recognize human leukocyte antigen (HLA) class I molecules as their ligands; *KIR2DL1* recognizes *HLA*-C group 2 (*HLA-C2*) allotypes having lysine at amino acid position 80, whereas *KIR2DL2* and KIR2DL3 recognize *HLA-C* group 1 (*HLA-C1*) allotypes having asparagine at amino acid position 80 [5]. *KIR2DL2* and *KIR2DL3* also recognize HLA-B*4601 acquiring the*-*C1 epitope by gene conversion [6]. Furthermore, *KIR3DL1* recognizes subsets of *HLA*-A and *HLA*-B allotypes having the *-*Bw4 epitope determined by amino acid positions 77-83 [7].

It has been well documented that certain KIR-HLA receptor-ligand combinations are associated with susceptibility to infectious diseases, such as HCV, as well as with disease progression and treatment response [8-15]. Recent reports have also identified a relationship between interleukin (IL) 28B gene polymorphisms and treatment and spontaneous resolution of HCV infection[16-19]. Dring et al. observed that the presence of *IL28B* gene polymorphisms and *KIR* genotypes synergized to increase the risk of chronic HCV infection[20], although this finding is under debate[21]. Suppiah et al. [22] recently reported that genotyping for *IL28B*, *HLA*-C, and *KIR* genes was useful for predicting HCV treatment response in patients of European descent. As these gene associations have not yet been studied in the Japanese population, we evaluated whether HLA-KIR interactions, in addition to an *IL28B* polymorphism, would influence the outcome of pegylated-interferon-α (PEG-IFN) and ribavirin therapy in Japanese patients with chronic hepatitis C. 

## Materials and Methods

### Ethics statement

 This study was approved by the ethical committee of Shinshu University School of Medicine, Matsumoto, Japan, and written informed consent was obtained from all participants. The study was conducted in accordance with the principles of the Declaration of Helsinki.

### Subjects

One hundred and fifteen consecutive IFN-treatment-naïve patients with chronic hepatitis C were enrolled in this study. All subjects were seen at Shinshu University Hospital or one of its affiliated hospitals. The clinical and demographic characteristics of our cohort are shown in Table 1. Diagnosis of chronic hepatitis C was based on previously reported criteria [23]: 1) presence of serum HCV antibodies and detectable viral RNA; 2) absence of detectable hepatitis B surface antigen and antibody to the human immunodeficiency virus; and 3) exclusion of other causes of chronic liver disease or a history of decompensated cirrhosis or HCC. Serum levels of HCV RNA were determined using Cobas Amplicor assays (sensitivity: 50 IU/mL; Roche Diagnostic Systems, Tokyo, Japan). HCV genotypes were determined using INNO-LiPA HCV II kits (Innogenetics, Gent, Belgium). Alanine aminotransferase (ALT), aspartate aminotransferase (AST), and other relevant biochemical tests were performed using standard methods[24]. Liver fibrosis was assessed using the AST to platelet ratio index (APRI) in this study. APRI has been recognized as a noninvasive test to estimate the degree of liver fibrosis in chronic liver disease with HCV infection[25]. APRI was calculated for all study subjects as follows: AST/upper limit of normal (45 IU/L) × 100/platelet count (10^9^/L). Patients received PEG-IFN-α2b (Pegintron; MSD KK, Tokyo, Japan; 1.5 μg/kg of body weight by subcutaneous injection once per week) and ribavirin (Rebetol; MSD KK; 600-1000 grams daily, according to body weight) for 48 weeks, as described previously[26]. Patients achieving a sustained HCV response were defined as those whose serum HCV RNA was undetectable 24 weeks after completing therapy. Patients who did not meet this criterion, who included non-responders and relapsers, were regarded as treatment failures. 

**Table 1:** Clinical features of sustained and non-sustained virological response patients with chronic hepatitis C.

Characteristic	All	SVR	Non-SVR	*P*
	(n = 115)	(n = 56)	(n = 59)	
Age (yr)	60 (24 - 80)	59 (25 - 80)	60 (24 - 75)	0.43
Male	66 (57)	34 (61)	32 (54)	0.48
Alanine aminotransferase (IU/L)	46 (17 - 389)	48 (17 - 389)	45 (17 - 309)	0.81
Aspartate aminotransferase (IU/L)	43 (17 - 246)	42 (17 - 231)	43 (17 - 246)	0.49
White blood cells (/μL)	4410 (2280 - 8240)	4740 (2700 - 8170)	4070 (2280 - 8240)	0.011
Hemoglobin (g/dL)	14.4 (9.2 - 18.2)	15.1 (11.0 - 18.2)	13.9 (9.2 - 17.4)	0.002
Platelet count (10^4^/μL)	15.9 (6.7 - 33.6)	16.6 (8.3 - 26.2)	15.6 (6.7 - 33.6)	0.30
APRI	0.89 (0.21 - 5.40)	0.59 (0.22 - 5.40)	0.66 (0.21 - 5.06)	0.41
HCV RNA (log_10_ IU/mL)	6.4 (5.0 - 7.3)	6.1 (5.0 - 6.8)	6.5 (5.0 - 7.3)	< 0.001

Data are expressed as median (range) or n (%) as appropriate. SVR, sustained virological response; HCV, hepatitis C virus

### HLA, KIR, and IL28B (rs8099917) Genotyping

 Genomic DNA was isolated from whole blood samples using QuickGene-800 assays (Fujifilm, Tokyo, Japan). We genotyped *HLA-*B, *HLA-*C, and *KIR* using a Luminex multi-analyzer profiling system with a LAB type® HD and KIR SSO genotyping kit (One Lambda, Inc., Canoga Park, CA), which is based on PCR sequence-specific oligonucleotide probes[27]. Subjects were identified as having the B/x or A/A genotype as defined previously[28]. Genotypes for the centromeric (*Cen*) and telomeric (*Tel*) parts of the *KIR* locus were determined according to the presence or absence of one or more B haplotype-defining *KIR* genes. Thus, *Cen-A1* and *Tel-A1* were the centromeric and telomeric motifs, respectively, of the canonical *A KIR* haplotype in the present study, *Cen-B1* and *Cen-B2* were alternative centromeric motifs of common *B KIR* haplotypes, and *Tel-B1* was the common telomeric motif of *B* haplotypes[29]. For much of this analysis, *Cen-B1* and *-B2* were grouped together as *Cen-B*, whereas *Cen-A1* was shortened to *Cen-A* and *Tel-A1* to *Tel-A*, as reported previously[30,31]. Genotyping of an *IL28B* SNP (rs8099917) was performed using a TaqMan 5’ exonuclease assay with primers supplied by Applied Biosystems[32]. Probe fluorescence signals were detected using a TaqMan assay for Real-Time PCR (7500 Real Time PCR System, Applied Biosystems) according to the manufacturer’s instructions.

### Statistical Analysis

The Mann-Whitney *U* test was employed to analyze continuous variables. Pearson’s chi-squared test was used for the analysis of categorical data. We adopted Fisher’s exact test when the number of subjects was less than 5. The Bonferroni correction for multiple testing was applied to our data of KIR-HLA combinations using the number of comparisons performed by our primary factors of interest in Table 2 (i.e., 8 tests = 4 combinations × 2 comparisons between two groups). A P value of < 0.05 was considered to be statistically significant. Association strength was estimated by calculating the odds ratio (OR) and 95% confidence interval (CI). Our model was checked by regression diagnostic plots to verify normality, linearity of data, and constant variance. Stepwise logistic regression analysis with a forward approach was performed to identify independent factors associated with an SVR after continuous variables were separated into 2 categorical variables by each median value. Statistical analyses were performed using SPSS software version 21.0J (IBM, Tokyo, Japan). Sensitivity, specificity, positive predictive value (PPV), and negative predictive value (NPV) were calculated to determine the reliability of the predictors of therapy response.

**Table 2:** Frequency of *IL28B* genotype, *KIR3DL1*/*HLA-Bw4*, and *KIR2DL2*/*HLA-C1* combinations in 56 patients with a sustained virological response (SVR) and 59 patients with a non-SVR to pegylated interferon and ribavirin therapy of chronic hepatitis C.

*KIR3DL1/HLA-Bw4*	*KIR2DL2/HLA-C1*	SVR	Non-SVR	P (Pc)	OR (95% CI)
		(n = 56)	(n = 59)		
+/+	+/+	5 (9%)	7 (12%)	0.61	
+/+	Other	31 (55%)	19 (32%)	0.012 (0.1)	2.61 (1.22 - 5.58)
Other	+/+	1 (2%)	10 (17%)	0.014 (0.12)	0.09 (0.01 - 0.72)
Other	Other	19 (34%)	23 (39%)	0.57	
*IL28B*	*KIR3DL1/HLA-Bw4*	SVR	Non-SVR	P (Pc)	OR (95% CI)
		(n = 56)	(n = 59)		
TT	+/+	27 (48%)	13 (22%)	0.003 (0.024)	3.29 (1.47 - 7.39)
TT	Other	17 (30%)	14 (24%)	0.42	
TG/GG	+/+	9 (16%)	13 (22%)	0.42	
TG/GG	Other	3 (5%)	19 (32%)	0.00062 (0.0005)	0.12 (0.03 - 0.43)
*IL28B*	*KIR2DL2/HLA-C1*	SVR	Non-SVR	P (Pc)	OR (95% CI)
		(n = 56)	(n = 59)		
TT	Other	38 (68%)	18 (31%)	0.000062 (0.0005)	4.81 (2.19 - 10.58)
TT	+/+	6 (11%)	9 (15%)	0.47	
TG/GG	Other	12 (21%)	24 (41%)	0.026 (0.21)	0.40 (0.17 - 0.91)
TG/GG	+/+	0 (0%)	8 (14%)	0.013 (0.1)	-

Data are expressed as n (%).

## Results

### Patient Characteristics and Treatment Outcome

 All patients in our test cohort were infected with HCV genotype 1b. Of the 115 patients receiving PEG-IFN-α2b and ribavirin therapy, 56 (49%) achieved an SVR. The remaining 59 patients were non-responders, 28 of whom experienced a relapse and 31 who were null responders. The median white blood cell count (*P* = 0.011) and hemoglobin value (*P* = 0.002) in the SVR group were significantly higher than those in the non-SVR group prior to treatment. HCV viral load at baseline was significantly associated with treatment outcome (*P* < 0.001).

### Association of HLA and KIR with a Sustained Virological Response

**Figure 1:**
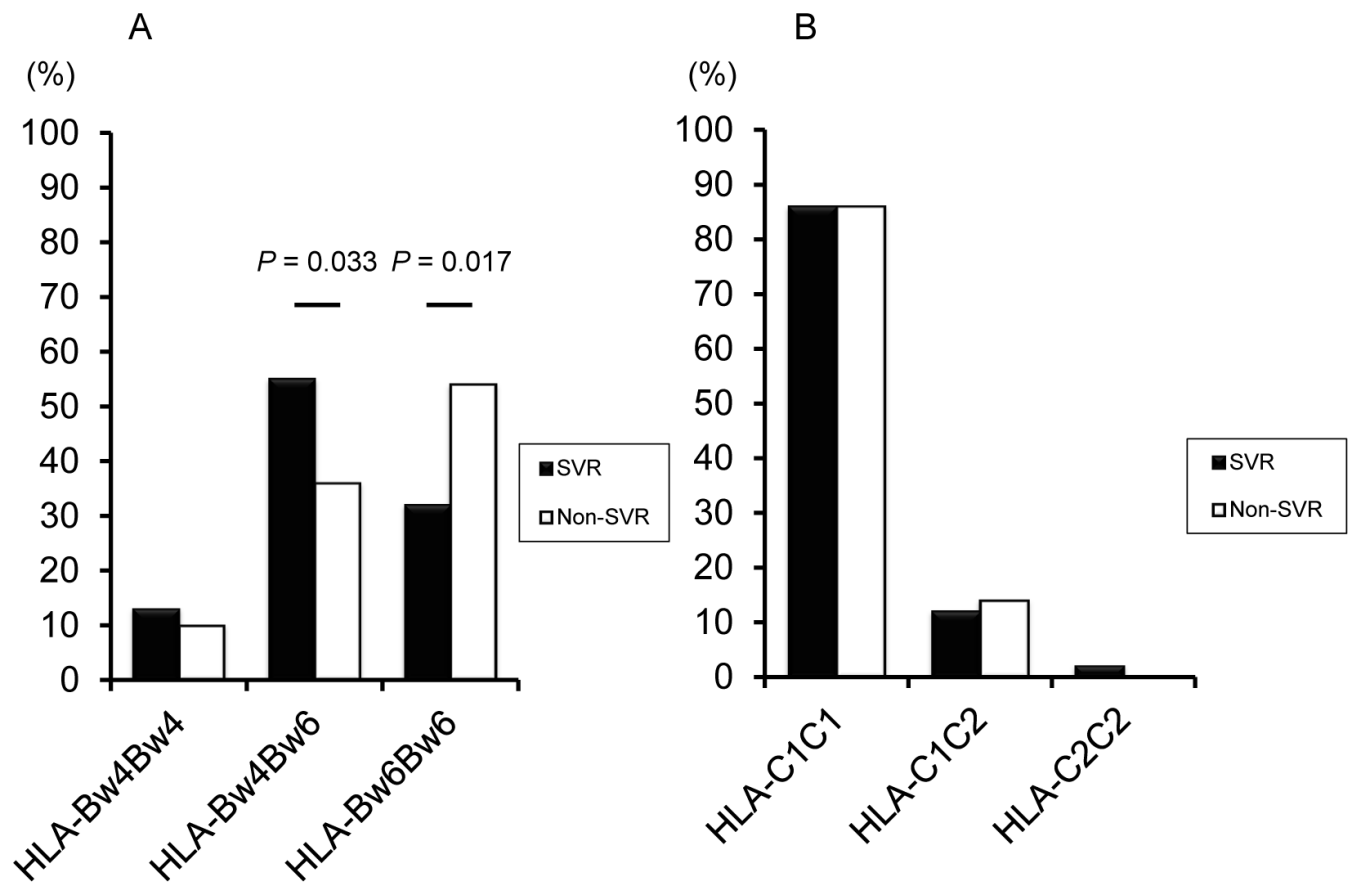
Frequency of *HLA*-*Bw* and -C alleles in 56 patients with a sustained virological response (SVR) and 59 patients with a non-SVR to pegylated interferon and ribavirin therapy of chronic hepatitis C.

 We first determined the frequency of *HLA*-*Bw* and *HLA-C* alleles in SVR and non-SVR patients (Figure 1). The frequency of *HLA-Bw4Bw6* in responders was significantly higher than that in non-responders (55% [31/56] vs. 36% [21/59]; *P* = 0.033; OR = 2.24, 95% CI = 1.06 - 4.75). Conversely, patients with the *HLA-Bw6* homozygote had a higher non-SVR rate (32% [18/56] vs. 54% [32/59]; *P* = 0.017; OR = 0.40, 95% CI = 0.19 - 0.85). Overall, *HLA-Bw4* was associated with an SVR among patients (68% [38/56] vs. 46% [27/59]; *P* = 0.017; OR = 2.50, 95% CI = 1.17 - 5.35). The frequencies of HLA-C were not statistically significant. We further checked whether particular HLA-Bw or HLA-C alleles were beneficial to treatment outcome. The HLA-B*35:01 allele was more frequently found in patients with an SVR than in those without (13% [15/102] vs. 4% [5/118]; *P* = 0.014 [*Pc* = 0.36]; OR = 3.49, 95% CI = 1.23 - 9.97).

**Figure 2:**
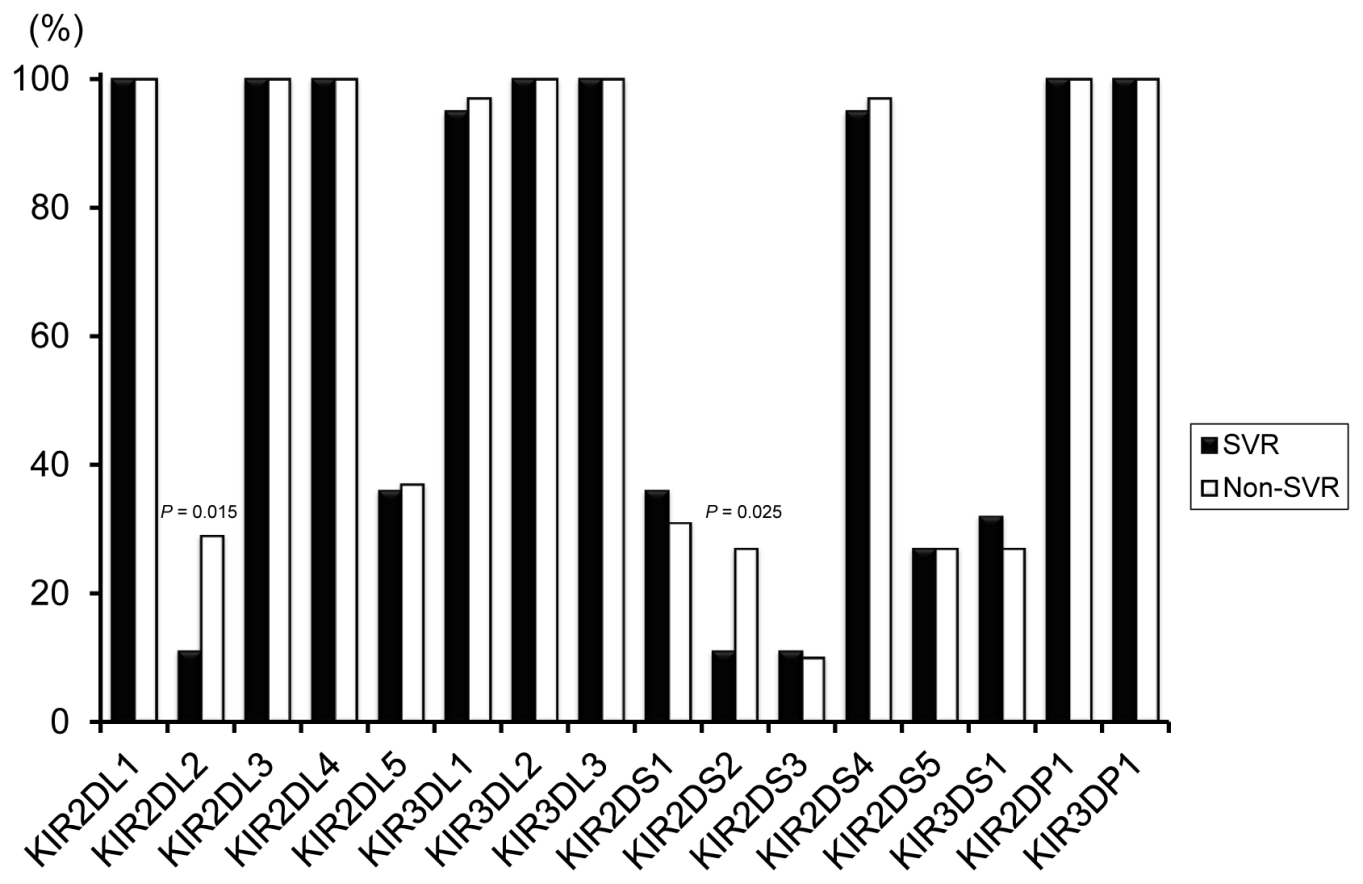
Frequency of each *KIR* gene in 56 patients with a sustained virological response (SVR) and 59 patients with a non-SVR to pegylated interferon and ribavirin therapy of chronic hepatitis C.

 The distribution of *KIR* genes and their association with treatment outcome are shown in Figure 2. No statistically significant differences were found for any allele combination apart from *KIR2DL2* and *KIR2DS2*; patients with these genes had significantly decreased SVR frequencies compared with those without (*P* = 0.015 [*Pc* = 0.48]; OR = 0.30, 95% CI = 0.11 - 0.82 and *P* = 0.025 [*Pc* = 0.8]; OR = 0.32, 95% CI = 0.12 - 0.90, respectively).

**Figure 3:**
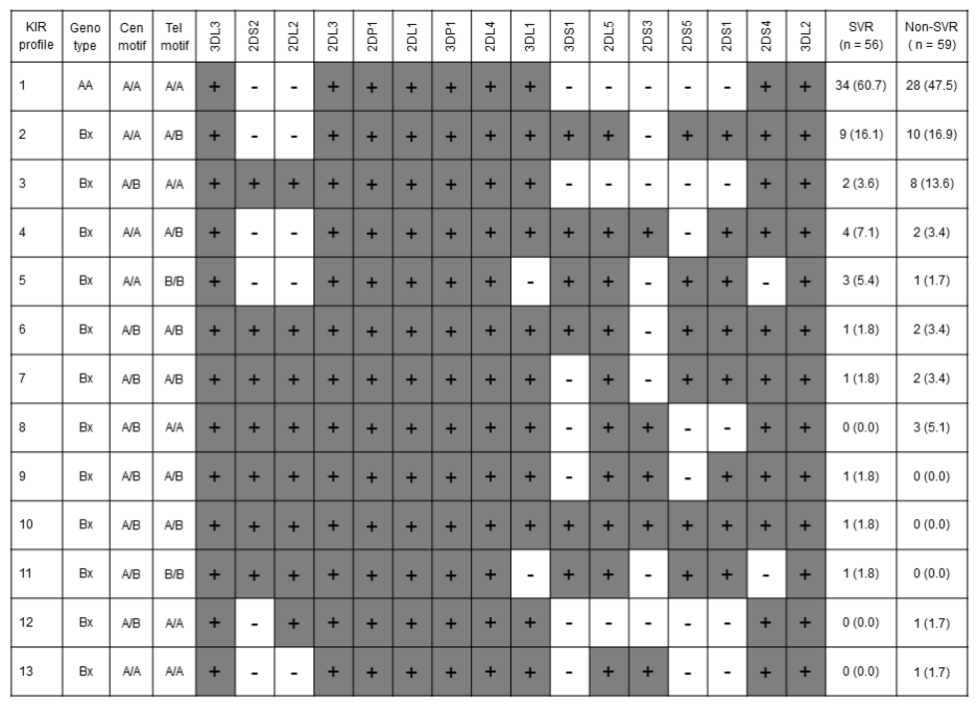
*KIR* gene profile frequencies in 56 patients with a sustained virological response (SVR) and 59 patients with a non-SVR to pegylated interferon and ribavirin therapy of chronic hepatitis C. Numerical data represent the number of individuals (%). The presence of *KIR* genes is indicated by gray shading. Cen, centromeric; Tel, telomeric.

*KIR* genotype profiles were determined by the presence or absence of each *KIR* locus in patients (Figure 3). Since strong linkage disequilibrium is a prominent feature in the *KIR* region, *KIR* gene profiles were classified based on *Cen* and *Tel* motifs. When we evaluated SVR according to genotype and *Cen* and *Tel* frequencies, we observed that virologic clearance with *Cen-A/A* was significantly higher than that without (54% [50/92] vs. 26% [6/23], *P* = 0.015; OR = 3.37, 95% CI = 1.22 - 9.33). There were no significant differences regarding *AA* genotype and *Tel*.

 We next analyzed combinations of activation/inhibitory *KIR*s and their *HLA* ligands for possible associations with an SVR. Among the combinations of *KIR3DL1-HLA-Bw4*, *KIR2DL2*-*HLA-C1*, and KIR2DL1-*HLA*-C2, patients who carried the inhibitory *KIR3DL1* receptor and its ligand *HLA-Bw4* had a significantly higher response rate than those without *KIR3DL1* or *HLA-Bw4* (58% [36/62] vs. 38% [20/53]; *P* = 0.030 [*Pc* = 0.12]; OR = 2.29, 95% CI = 1.08 - 4.84). In contrast, the *KIR2DL2*-*HLA-C1* combination resulted in a significantly lower SVR rate (26% [6/23] vs. 54% [50/92]; *P* = 0.015 [*Pc* = 0.06]; OR = 0.30, 95% CI = 0.11 - 0.82). Although several studies have found that *KIR2DL3-HLA-C1* carriers are associated with treatment-induced and spontaneous clearance of HCV in Caucasians, no such association was found in our cohort (data not shown).

 Patients with *KIR3DL1*-*HLA-Bw4* but without *KIR2DL2*-*HLA-C1* had a higher SVR rate (55% [31/56] vs. 32% [19/59]; *P* = 0.012 [*Pc* = 0.1]; OR = 2.61, 95% CI = 1.22 - 5.58) (Table 2). Conversely, the frequency of the *KIR2DL2*-*HLA-C1* positive, but *KIR3DL1*-*HLA-Bw4* negative condition was significantly higher in non-responders (17% [10/59] vs. 2% [1/56]; P = 0.014 [*Pc* = 0.12]; OR = 0.09, 95% CI = 0.01 - 0.72). 

### Prediction of a Sustained Virological Response by KIR-HLA and IL28B

 Examination of the *IL28B* rs8099917 SNP in our cohort revealed significant differences in SVR frequencies. The SVR rate in patients with the *IL28B* TT genotype was significantly higher in those with TG or GG genotypes (62% [44/71] vs. 27% [12/44), *P* = 0.0003; OR = 4.35, 95% CI = 1.92 - 9.85). In subjects with *IL28B* TT and *KIR3DL1*-*HLABw4*, virologic clearance was significantly increased over other combinations (68% [27/40] vs. 39% [29/75]; *P* = 0.003 [*Pc* = 0.024]; OR 3.29, 95% CI = 1.47 - 7.39).

We next evaluated several factors found in association with an SVR to PEG-IFN and ribavirin therapy for independence by logistic regression analysis. Fifty-six responders were compared with 59 non-responders by means of a forward stepwise likelihood ratio logistic regression method; estimated OR coefficients, 95% CI, and *P* values are summarized in Table 3 for the variables that remained in equation at the last step. *IL28B* TT genotype (*P* = 0.00009; OR = 6.87, 95% CI = 2.62 - 18.01), *KIR2DL2-HLA-*C1 (*P* = 0.014; OR = 0.24, 95% CI = 0.08 - 0.75), white blood cell count ≥ 4410/μL (*P* = 0.009; OR = 3.32, 95% CI= 1.35 - 8.16), and *KIR3DL1*-*HLA-Bw4* (*P* = 0.008; OR = 3.32, 95% CI = 1.37 - 8.05) were all identified as independent parameters that significantly influenced an SVR. 

**Table 3:** Logistic regression analysis of variables contributing to a sustained virological response to pegylated interferon and ribavirin.

Factor	Odds ratio	95% confidence interval	*P*
*IL28B* TT genotype	6.87	2.62 - 18.01	0.00009
*KIR2DL2*/*HLA-*C1	0.24	0.08 - 0.75	0.014
White blood cells ≥ 4410/μL	3.32	1.35 - 8.16	0.009
*KIR3DL1*/*HLA-Bw4*	3.32	1.37 - 8.05	0.008

Only variables achieving statistical significance (*P* < 0.05) in multivariate logistic regression analysis are shown.

 The frequency of the *IL28B* TT genotype with *KIR3DL1*-*HLA-Bw4* in responders was significantly higher than in non-responders (48% [27/56] vs. 22% [13/59]; *P* = 0.003 [*Pc* = 0.024]; OR = 3.29, 95% CI = 1.47 - 7.39) (Table 2). Patients with the *IL28B* TT genotype without *KIR2DL2*-*HLA-C1* had a significantly higher SVR rate (68% [38/56] vs. 31% [18/59]; *P* = 0.000062 [*Pc* = 0.0005]; OR = 4.81, 95% CI = 2.19 - 10.58). The frequency of a non-SVR was significantly higher in patients with the *IL28B* non-TT genotype both with and without *KIR2DL2*-*HLA-C1* (14% [8/59] vs. 0% [0/8]; *P* = 0.013 [*Pc* = 0.1] and 41% [24/59] vs. 21% [12/56]; *P* = 0.026 [*Pc* = 0.21]; OR = 0.40, 95% CI = 0.17 - 0.91, respectively). The ability to predict an SVR by *IL28B* genotype and *KIR3DL1*-*HLA-Bw4* and *KIR2DL2*-*HLA-C1* was next evaluated. Corresponding values for sensitivity, specificity, PPV, and NPV are listed in Table S1 in File S1. A combination of the *IL28B* TT genotype and *KIR3DL1*-*HLA-Bw4* demonstrated high predictive specificity (78%), as did the combination of *IL28B* TT genotype and *KIR2DL2*-*HLA-C1* (86%).

 Lastly, we analyzed combinations of the three factors of *IL28B* genotype, *KIR3DL1*-*HLA-Bw4*, and *KIR2DL2*-*HLA-C1* for prediction of treatment outcome (Table S2 in File S1). The frequencies of *IL28B* TT, *KIR2DL2*-*HLA-C1-*negative, with and without *KIR3DL1*-*HLA-Bw4* were significantly higher among responders (38% [21/56] vs. 19% [11/59]; P = 0.024 [Pc = 0.29]; OR = 2.62, 95% CI = 1.12 - 6.12 and 30% [17/56] vs. 12% [7/59]; P = 0.015 [Pc = 0.18]; OR = 3.24, 95% CI = 1.22 - 8.57, respectively).

## Discussion

 The present study examined *HLA*, *KIR*, and *IL28B* gene variant associations with an SVR following PEG-IFN and ribavirin therapy in Japanese patients with chronic hepatitis C. We found a significant association of *HLA-Bw* alleles with treatment outcome, although the frequency of *HLA-C* alleles did not differ significantly between responders and non-responders. Functional analyses have demonstrated that NK cells in *HLA-C1C1* subjects exhibit a more rapid and stronger antiviral response that those in *HLA*-*C2C2* subjects due to differing responses of *HLA*-*C*-inhibited NK subsets[33]. *HLA*-*C2C2* homozygousity is strongly associated with treatment failure in HCV patients of European ancestry [11,22], but we could not assess its role in our study because this genotype was found in only 1 of 115 patients. 

 We uncovered a significant association between the presence of *KIR2DL2* or *KIR2DS2* and lower SVR rates. Several reports have shown that *KIR2DL3-HLAC1* in Caucasians [11,22] and *KIR2DL5* in Brazilians [34] are associated with treatment outcome of antiviral therapy. Since our results showed no such statistical significances, these conflicting interpretations may reflect differences in patient selection, genetic background, sample size, and/or treatment regimen. Further studies are required to clarify this discrepancy in the Japanese population.

 A study by Dring et al. examined *KIR* haplotypes in patients with HCV infection and showed that a centromeric *KIR* haplotype was increased in chronic HCV infection as compared with resolved cases [20]. We therefore determined *KIR* haplotypes and *Cen-A/B* and *Tel-A/B* in our patients as well, and found an interesting association between *Cen-A/A* and an SVR to antiviral therapy (*P* = 0.015; OR 3.37). Since *Cen-A/B* is determined by *KIR2DL3* and *KIR2DS2* and/or *KIR2DL2*, this finding is consistent with our results demonstrating a relationship between *KIR2DS2* and *KIR2DL2* genotypes and treatment failure. 

 The most significant finding in this study was the association between KIR-HLA receptor-ligand pairings and treatment outcome in chronic hepatitis C. Among the inhibitory KIR-HLA receptor-ligand pairs, patients with *KIR3DL1*-*HLA-Bw4* exhibited a significantly higher SVR rate when compared to those without this pair (*P* = 0.03; OR 2.29). Conversely, virologic clearance in patients with *KIR2DL2-HLA-C1* was significantly lower than in those without (*P* = 0.015; OR = 0.30). Stratification analysis of the 4 groups of *KIR3DL1-HLA-Bw4* (presence or absence) and *KIR2DL2-HLA-C1* (presence or absence) revealed a higher frequency of responders with *KIR3DL1-HLA-Bw4* presence*, KIR2DL2-HLA-C1* absence compared with those possessing *KIR2DL2-HLA-C1* presence*, KIR3DL1-HLA-Bw4* absence (62% vs. 9%; *P* = 0.0044; OR =16.32). When these KIR-HLA pairs were both either positive or negative, SVR rates were similar at 42% and 45%, respectively. Together with the results of logistic regression analysis, we clearly showed that *KIR3DL1*-*HLA-Bw4* was positively associated with an SVR (OR = 3.32) and that *KIR2DL2-HLA-C1* had a negative association (OR = 0.24) with treatment outcome. As almost one half of the Japanese population have the functional *KIR3DL1-HLA-Bw4* combination, this inhibitory receptor-ligand interaction is potentially important in understanding NK cell diversification. The NK-cell surface expression of KIR3DL1 is higher in individuals having Bw4 than in those lacking it [35]. Therefore, these cells might be more weakly controlled by inhibitory signals than other NK cells, more easily activated by viral infection, and more readily promoted for cytolysis and IFN-gamma production.

 This study confirmed that the *IL28B* TT genotype is a strong predictor of an SVR in Japanese patients[18,32]. Furthermore, SVR frequencies were positively correlated with a combination of the *IL28B* TT genotype and *KIR3DL1*-*HLA-Bw4* (*P* = 0.0019) and negatively associated with the *IL28B* TT genotype and *KIR2DL2-HLA-C1* (*P* = 0.0067). These combinations were also highly specific for virologic response prediction. In light of these findings, patients with poor expected treatment outcome may be advised to wait for the use of combinations of direct acting antiviral agents[36]. Akuta et al. reported that a combination of amino acid substitutions in the core region of HCV and *IL28B* genotype was a useful predictor of PEG-IFN, ribavirin, and telaprevir therapy results in Japan[37]. Since we could not collect sera before treatment for all patients, we were not able to assess the effect of amino acid substitutions in the HCV core region. Furthermore, interferon-free combinations of direct-acting antiviral agents have become an area of considerable clinical interest. Chu et al. have reported that IL28B genotype appears to affect early viral kinetics in patients with chronic hepatitis C receiving interferon-free treatment [38]. Recently, two groups have discovered *IFN* lambda *4* (*IFNL4*), a new gene that may account for associations of spontaneous and IFN-based treatment clearance of HCV [39,40]. The IFN-λ 4 protein is generated by individuals who carry the ∆G allele of the ss469415590 variant, and the presence of this protein is strongly associated with impaired clearance of HCV. Linkage disequilibrium is strong between the *IFNL4-∆G* allele and the unfavorable rs12979860-T allele (*IL28B*) in subjects of European or Asian ancestry, whereas this linkage disequilibrium is moderate in individuals of African ancestry [39]. We have confirmed that the linkage disequilibrium between the *IFNL4-∆G* allele and *IL28B* SNP (rs8099917) is high and that the *IFNL4-∆G* allele is strongly associated with treatment failure of PEG-IFN and ribavirin therapy in patients with Japanese chronic hepatitis C [41]. Hence, the clinical impacts of HLA-KIR genetic variants, IL28B genotype, and the IFNL4 allele should be explored.

 In conclusion, the present study showed significant associations of *KIR3DL1-HLA-Bw4, KIR2DL2-HLA-C1*, and *IL28B* combinations with an SVR to PEG-IFN and ribavirin therapy in Japanese patients with genotype 1 HCV. The clinical significance of *IL28B* genotyping combined with HLA/KIR pairs to predict treatment outcome warrants further validation for triple therapy.

## Supporting Information

File S1 **Table S1, Sensitivity, specificity, and predictive values of IL28B TT genotype and KIR3DL1/HLA-Bw4 or KIR2DL2/HLA-C1 for a sustained virological response in 115 patients with chronic hepatitis C.** Data are expressed as % (n). PPV, positive predictive value; NPV, negative predictive value. Table **S2**, Frequency of ***IL28B*** genotype and ***KIR3DL1*/*HLA-Bw4*** and ***KIR2DL2*/*HLA-C1*** combinations in 56 patients with a sustained virological response (SVR) and 59 patients with a non-SVR to pegylated interferon and ribavirin therapy of chronic hepatitis C. Data are expressed as n (%).
(DOC)
