# Supplementary material for: KIR, HLA, and IL28B Variant Predict Response to Antiviral Therapy in Genotype 1 Chronic Hepatitis C Patients in Japan
Source: PLoS One. 2013 Dec 12;8(12):e83381. doi: 10.1371/journal.pone.0083381 (PMC3861489; doi:10.1371/journal.pone.0083381)
Supplement: File S1 — Table S1, Sensitivity, specificity, and predictive values of IL28B TT genotype and KIR3DL1/HLA-Bw4 or KIR2DL2/HLA-C1 for a sustained virological response in 115 patients with chronic hepatitis C. Data are expressed as % (n). PPV, positive predictive value; NPV, negative predictive value. Table S2, Frequency of IL28B genotype and KIR3DL1/HLA-Bw4 and KIR2DL2/HLA-C1 combinations in 56 patients with a sustained virological response (SVR) and 59 patients with a non-SVR to pegylated interferon and ribavirin therapy of chronic hepatitis C. Data are expressed as n (%). (DOC) [file pone.0083381.s001.doc]

**Table S1. Sensitivity, specificity, and predictive values of IL28B TT genotype and KIR3DL1/HLA-Bw4 or KIR2DL2/HLA-C1 for a sustained virological response in 115 patients with chronic hepatitis C**

|  | Sensitivity | Specificity | PPV | NPV |
| --- | --- | --- | --- | --- |
| (1) IL28B TT genotype | 79 (44/56) | 54 (32/59) | 62 (44/71) | 73 (32/44) |
| (2) KIR3DL1+/HLA-Bw4+ | 63 (35/56) | 56 (33/59) | 57 (35/61) | 61 (33/54) |
| (3) KIR2DL2+/HLA-C1+ | 11 (6/56) | 75 (44/59) | 29 (6/21) | 47 (44/94) |
| (1) and (2) | 46 (26/56) | 78 (46/59) | 67 (26/39) | 61 (46/76) |
| (1) and/or (2) | 95 (53/56) | 32 (19/59) | 57 (53/93) | 86 (19/22) |
| (1) and (3) | 11 (6/56) | 86 (51/59) | 43 (6/14) | 46 (51/111) |
| (1) and/or (3) | 79 (44/56) | 42 (25/59) | 56 (44/78) | 68 (25/37) |

Data are expressed as % (n).

PPV, positive predictive value; NPV, negative predictive value.

**Table S2. Frequency of *IL28B* genotype and *KIR3DL1*/*HLA-Bw4* and *KIR2DL2*/*HLA-C1* combinations in 56 patients with a sustained virological response (SVR) and 59 patients with a non-SVR to pegylated interferon and ribavirin therapy of chronic hepatitis C.**

| *IL28B* | *KIR3DL1/HLA-Bw4* | *KIR2DL2/HLA-C1* | SVR  (n = 56) | Non-SVR  (n = 59) | *P* (*Pc*) | OR (95% CI) |
| --- | --- | --- | --- | --- | --- | --- |
| TT | +/+ | Other | 21 (38%) | 11 (19%) | 0.024 (0.29) | 2.62 (1.12-6.12) |
| TT | Other | +/+ | 1 (2%) | 7 (12%) | 0.08 |  |
| TT | +/+ | +/+ | 5 (9%) | 2 (3%) | 0.39 |  |
| TT | Other | Other | 17 (30%) | 7 (12%) | 0.015 (0.18) | 3.24 (1.22-8.57) |
| TG/GG | +/+ | Other | 9 (16%) | 8 (14%) | 0.70 |  |
| TG/GG | Other | +/+ | 0 (0%) | 3 (5%) | 0.26 |  |
| TG/GG | +/+ | +/+ | 0 (0%) | 5 (9%) | 0.077 |  |
| TG/GG | Other | Other | 3 (5%) | 16 (27%) | 0.0039 (0.047) | 0.15 (0.04-0.56) |

Data are expressed as n (%).
